# A Survey on Hong Kong Secondary School Students' Knowledge of Emergency Management of Dental Trauma

**DOI:** 10.1371/journal.pone.0084406

**Published:** 2014-01-06

**Authors:** Cecilia Young, Kin Yau Wong, Lim K. Cheung

**Affiliations:** 1 Private Practice, Hong Kong; 2 Department of Biostatistics, University of North Carolina at Chapel Hill, Chapel Hill, North Carolina, United States of America; 3 BDS (Glasgow), FFDRCS (Ireland), FDSRCPS (Glasgow), FRACDS (Australia), FRACDS (OMS) (Australia), PhD (HK), Hon FDSRCS (Edin), FHKAM (Dental Surgery), FCDSHK (Oral and Maxillofacial Surgery); 4 Chair Professor, Oral and Maxillofacial Surgery, Faculty of Dentistry, The University of Hong Kong, Hong Kong; University of South Australia, Australia

## Abstract

**Objectives:**

To investigate Hong Kong secondary school students' knowledge of emergency management of dental trauma.

**Method:**

A questionnaire survey on randomly selected secondary school students using cluster sampling.

**Results:**

Only 36.6% (209/571) of the respondents were able to correctly identify the appropriate place for treatment of dental injury. 55.2% of the respondents knew the suitable time for treatment. Only 24.7% of the respondents possessed the knowledge of how to correctly manage fractured teeth. Only 23.6% of them knew how to manage displaced teeth. 62.5% of them correctly answered that knocked-out deciduous teeth should not be replanted to the original position, but few of them (23.6%) knew that permanent teeth should be replanted. Moreover, 37.1% of the respondents correctly identified at least one of the appropriate media for storing a knocked-out tooth. First-aid training and acquisition of dental injury information from other sources were significant factors that positive responses from these questions would lead to higher scores.

**Conclusion:**

Hong Kong secondary school students' knowledge of emergency management of dental trauma is considered insufficient. An educational campaign in secondary schools dedicated to students is recommended. Prior first-aid training and acquisition of dental injury information from other sources positively relate to the level of knowledge. Dental trauma emergency management is recommended to be added to first-aid publications and be taught to students and health professionals.Trial Registration: Hong Kong Clinical Trial Centre

HKCTR-1344

## Introduction

The consequences of traumatic dental injuries range from tooth fractures to avulsion of the whole tooth. The prognosis of some of the dental injuries, especially avulsion, depends on early management [1].

Surveys on children and adolescents have shown that falls [2]–[14], sports activities [2], [3], [5], [7], [9], [12]–[14], collisions [3], [9], [11], [12], physical leisure activities [3], [7], [11]–[13], being struck by an object [4], [6], [11], [12] and traffic accidents [2], [5] are the major causes of traumatic dental injuries. Among them, fall is the main cause of traumatic dental injuries [4], [6]–[14].

Home [6], [7], [9]–[12] and school [6], [7], [9]–[11] are the most common places where traumatic dental injuries occur. If the patient or persons nearby can perform immediate management at the site of injury, the level of harm can be reduced.

It is important to study whether students know how to perform simple but essential emergency procedures before a patient suffering dental trauma reaches professional care. Evaluating the corresponding knowledge level of students is important for understanding the need for educational campaigns. This information can also assist health promoters in requesting additional resources.

Searching for the keywords “knowledge” and (“dental trauma” or “dental injur*” or “traumatic dental injur*”) on Pubmed and Ovid Medline prior to the present study only resulted in three papers on children and youngster which are related to knowledge of dental trauma [15]–[17]. The results were the same up to Aug 27, 2013. Among the three papers, Castilho et al. studied sixth grade primary school children [15]. Andersson et al. interviewed 7- to 15-year-old school children [16]. Biagi et al. studied an 8- to 15-year-old group [17]. These authors concluded that these children and youngsters' knowledge of dental trauma was insufficient. No such information on students' knowledge of emergency management of dental trauma in Hong Kong was available.

There were 663 secondary schools with a total of 454,244 students in Hong Kong in 2011. Children aged around 6 years enter primary schools from Primary 1 to Primary 6 (US Grade 1–6). Then, they attend secondary schools from Form 1 at around age 12 to Form 7 (US Grade 7–12 plus 1 year). We chose the secondary school students from Form 1 to Form 7 because they should be able to comprehend written questionnaires.

### Objectives

1. To investigate Hong Kong secondary school students' knowledge of emergency management of dental trauma.
2. To investigate whether the secondary school students consider themselves capable of distinguishing between permanent and deciduous teeth.

## Methods

### Ethical approval

The Institutional Review Board of the University of Hong Kong and Hospital Authority Hong Kong West Cluster approved this research project (UW11- 186, HKCTR-1344), which complied with the Declaration of Helsinki and ICH GCP guidelines. The protocol for this study is available as supporting information; see attached Protocol S1.

### Subjects

The target group of this study was secondary school students from Form 1 to Form 7 (US Grade 7–12 plus 1 year) in Hong Kong who read Chinese or English.

### Sample size calculation

For the calculation of sample size for the estimation of the proportion of students having score higher than a certain threshold level, we used a 95% confidence level with 10% precision. As the population variance was unknown, we adopted *P* = 50% to allow for the greatest variance. Assuming an infinite population size, we needed a sample size of 97. To account for the cluster sampling design, we multiplied a design effect of 1+(*m*−1) *ρ*, with *m* being the cluster size and *ρ* being the intracluster correlation (ICC). We set the cluster size to be 40 and assumed an ICC of 0.1. No published data on ICC under this setting could be found. However, in general practice studies, ICC takes values commonly between 0.01 and 0.05 [18], so 0.1 is a conservative estimate. We needed a sample size of 476, or a total of 12 schools. To account for possible blank or incomplete returned questionnaires, we aimed at recruiting an extra 20%, which resulted in 15 schools or 600 students.

### Questionnaire design

Chinese and English questionnaires were constructed. The questionnaire consisted of 2 sections with a total of 14 questions. The first section included basic demographic information, whether the respondents had received formal first-aid training and acquired dental trauma information and whether they considered themselves capable of distinguishing between permanent or deciduous teeth. The second part consisted of 6 questions concerning the knowledge of dental traumatic injuries. The same set of questions on dental knowledge was used in the survey for primary and secondary school teachers in Hong Kong from the same series of studies [19].

The questionnaire is provided as supporting information. For Q9 to Q13, 1 mark would be given for a correct answer, no mark would be given for “do not know” and 1 mark would be deducted for an incorrect answer. If multiple answers were chosen, 1 mark would be deducted for that question if an incorrect answer was chosen. There were three correct answers for Q14 as the media for storing avulsed teeth. As avulsion is the most serious dental trauma, it is necessary to manage the emergency correctly right at the scene in order to save the avulsed teeth. The more storing media a person is aware of, the more likely he/she would be able to find one in time for keeping the vitality of the periodontal cells on the root surface of an avulsed tooth, thus improving the prognosis. Therefore, 1 mark would be given for a correct answer but 1 mark would be deducted for an incorrect answer. Multiple answers were allowed.

### Validity and reliability of questionnaire

The questionnaire was pilot tested with 59 students. Face validity was established by expert opinion; length and whether secondary school students could comprehend it were pre-tested before adoption.

In the reliability test, a group of 39 students from a local secondary school was recruited, and each was asked to complete the questionnaire. After two weeks, the group was asked to complete the same questionnaire again. All students returned the second questionnaire.

To assess the test-retest reliability, paired *t*-tests were conducted. For each question, the null hypothesis was that there was no significant difference between the scores of the first and second questionnaires. The level of significance was 5%. The Pearson's product moment correlation coefficient was also calculated for each question. A correlation coefficient closer to 1 indicates a higher reliability, as it represents a higher correlation between the scores of the first and second questionnaires. The results are presented in Table 1.

**Table 1:** Test-Retest Results.

	Original		Retest		Within person differences (Retest – Test)	Correlation
	Mean	SD*	Mean	SD	Mean (95% CI)	
Q9	−0.38	0.85	−0.31	0.86	0.08 (−0.04,0.19)	0.91
Q10	0.23	0.90	0.28	0.89	0.05 (−0.05,0.16)	0.94
Q11	−0.05	0.86	−0.05	0.86	0 (0,0)	1
Q12	−0.44	0.75	−0.41	0.75	0.03 (−0.03,0.08)	0.98
Q13	0.31	0.80	0.44	0.85	0.13 (−0.004,0.26)	0.88
Q14	−0.08	0.98	−0.03	0.99	0.05 (−0.02,0.12)	0.97
Total	−0.41	2.12	−0.08	2.08	0.33 (0.07, 0.59)	0.93

SD =  Standard Deviation.

All differences of the scores were not significantly different from zero at 5% significance as all the 95% confidence intervals included 0. However, the confidence interval for the total score did not include zero. The correlations were generally high, at levels around 0.9.

### Recruitment and implementation

We sent invitations to schools in batches starting on May 13, 2011 until the desired sample size was reached. Each batch consisted of 15 schools (5 for Forms 1–3, 5 for Forms 4 – 5 and 5 for Forms 6 – 7), which were randomly selected from the list of secondary schools obtained from the Educational Bureau using simple random sampling. An invitation pack sent to each selected school included the invitation letter, a school consent form, 50 individual guardian's consent forms, and 50 questionnaires.

After four batches of mailings, adding up to 60 schools, were sent, 14 schools returned the school consent forms, individual guardian's consent forms and the filled questionnaires. Invitation packs were sent to another 2 schools. On Oct 10, 2011, one more school returned the filled materials.

We requested each school to invite one or more classes with at least 40 students. We asked them to photocopy the guardian's consent forms and questionnaires if necessary. A teacher in charge from each school was responsible for obtaining consent forms and the distribution, supervision and collection of questionnaires. The teacher in charge made clear that the whole procedure was a survey instead of an examination and that there should be no discussion on the questionnaire.

The participating students could withdraw from the study at any time. This was mentioned in the invitation letter and in both consent forms.

### Statistical analysis

For each question concerning knowledge of dental trauma, the number and proportion of respondents who chose the correct answer(s) are reported.

To investigate the effects of the demographic and education backgrounds on the knowledge, a multiple linear regression on the total score was conducted, with independent variables (fixed effects) gender, age, form, receipt of first-aid training, dental injury content in first-aid training, confidence in distinguishing the type of teeth and acquisition of dental injury information from other sources. The school effect was considered as a random effect, which acted addictively to the total score. The school-specific random effect was normally distributed with zero mean and unknown variance.

The two variables, receipt of first-aid training and the dental injury content in first-aid training, were jointly represented by two dummy variables, *X_5_* and *X_6_*. The definitions were: *X_5_* = 1 if the respondent received first-aid training without dental injury content, and *X_5_* = 0 if the respondent had not received first-aid training. *X_6_* = 1 if the respondent had received first-aid training with dental injury content, and *X_6_* = 0 if the respondent had not received first-aid training.

The thresholds of all the statistical tests were set at 5% level of significance. The statistical analyses were performed using a computer programme (JMP version 9.0.2., SAS Institute Inc., USA).

## Results

We collected 601 questionnaires between May 24 and Oct 10, 2011. After removing those with missing entries in the first section of demographics and background information, 571 questionnaires were left for statistical analysis. They were from 15 schools, with each school having an average of 38 students. The summary statistics of the respondents' background information collected are tabulated in Table 2. 60.1% of the respondents were female and 53.9% of them were within the age range 14 – 16. 43.6% were from Form 1 – 3, 44.7% were from Form 4 – 5 and a small percentage of 11.7% were from F.6 – 7. 8.9% had received first-aid training, and only 3.0% (of the whole sample) had learnt about management of dental injury in the training program. 23.6% of the respondents considered themselves able to distinguish deciduous from permanent teeth. Finally, 29.8% of the respondents had acquired information about dental injury management from other sources besides from first-aid training program.

**Table 2:** Part 1 Demographics and characteristics of respondents.

	No. of respondents (*n* = 571)	Proportion of respondent (%)
**Gender**		
Male	228	39.9
Female	343	60.1
**Age**		
10 or below	1	0.2
11 – 13	132	23.1
14 – 16	308	53.9
17 – 19	121	21.2
20 or above	9	1.6
**Form**		
Form 1 – 3	249	43.6
Form 4 – 5	255	44.7
Form 6 – 7	67	11.7
**Received First-aid Training**		
Yes	51	8.9
No	520	91.1
**Learnt Dental Injury Management in First-aid Training**		
Yes	17	3.0
No	554	97.0
**Confident in Distinguishing Type of Teeth**		
Yes	135	23.6
No	436	76.4
**Read or heard dental injury information besides from First-aid Training**		
Yes	170	29.8
No	401	70.2

The percentages of responses to each question are tabulated in Table 3. The correct answer(s) for each question is (are) highlighted. Most of the questions were answered incorrectly, which is also reflected in Table 4. 36.6% of the respondents were able to identify the appropriate place for treatment of dental injury. 55.2% of the respondents answered correctly the suitable time for treatment. Only 24.7% of the respondents knew how to correctly manage fractured teeth. Only 23.6% of the respondents knew how to manage displaced teeth. 62.5% of the respondents correctly answered that knocked-out deciduous teeth should not be replanted to the original position, but only a comparatively much smaller proportion of them (23.6%) knew that avulsed permanent teeth should be replanted. Moreover, 37.1% of the respondents correctly specified at least one of the appropriate media for storing a knocked-out tooth.

**Table 3:** Options chosen in knowledge section.

	Proportion (%)*
**Q9 Place for Treatment**	
Go to the casualty in the nearest hospital on foot or by any transport	25.4
Call an ambulance; go to the casualty in the nearest hospital	15.6
Go to the nearest private doctor	5.3
Go to the patient's family doctor	2.5
Go to a dentist	44.7
Treat it by self	4.2
Others	0.4
Don’t Know	14.7
**Q10 Time for Treatment**	
At lunch or after school	1.8
After plenty of rest	2.8
Within 24 hours	7.7
Within 48 hours	1.8
When the parent or guardian is free to bring the patient for examination and treatment	2.5
Any time when the patient feels relax and want to have treatment	2.5
Within 4 hours	14
Immediately	57.1
Others	0.5
Don’t Know	14.2
**Q11 Immediate Management of Fractured Teeth**	
The fractured part is useless, ignore it	2.3
Try to find the fractured time, wrap it with gauze or tissue and bring it for examination and treatment	44.5
Put it in liquid medium and bring it for examination and treatment [20], [21]	24.7
Others	1.2
Don’t Know	27.5
**Q12 Immediate Management of Displaced Teeth**	
Do not touch, let it remains its new position	47.5
Try to put back to the original position [22]	4.7
Ask the patient to carefully clench one's teeth if possible [23]	19.6
Others	3.0
Don’t Know	25.9
**Q13i Should knocked-out baby teeth be put back to original position**	
Yes	4.2
No [24]–[26]	62.5
Don’t Know	33.3
**Q13ii Should knocked-out permanent teeth be put back to original position**	
Yes [21], [24], [25]	23.6
No	34.0
Don’t Know	42.4
**Q14 Medium for storing knocked-out teeth**	
The tooth is useless, do not spend time to find it or to work on it	5.1
Gauze or tissue	33.1
Cold milk [21], [24], [25]	18.7
Physiological saline [21], [24], [25]	24.2
Patient's saliva [21], [24], [25]	6.7
Tap water	5.4
Distilled water	17.5
A container or plastic bag in dry condition	6.1
Disinfectant solution	10.3
Others	1.1
Don’t Know	31.7

For Q9–12, 13i & 13ii, respondents should choose 1 answer but some respondents chose more than 1.

For Q14, multiple options could be chosen.

the sum of the proportions for a question may be larger than 1 as respondents might have chosen more than one answer. The numbers in bracket indicate the references.

**Table 4:** Score for each question in the knowledge section.

		Correct	Incorrect	Do not know
		*n* (%)	*n* (%)	*n* (%)
**Q9**	**Place for treatment**	209 (36.6)	276 (48.3)	86 (15.1)
**Q10**	**Time for treatment**	315 (55.2)	177 (31.0)	79 (13.8)
**Q11**	**Management of fractured teeth**	141 (24.7)	270 (47.3)	160 (28.0)
**Q12**	**Management of displaced teeth**	135 (23.6)	286 (50.1)	150 (26.3)
**Q13i**	**Management of avulsed baby teeth**	357 (62.5)	24 (4.2)	190 (33.3)
**Q13ii**	**Management of avulsed permanent teeth**	135 (23.6)	194 (34.0)	242 (42.4)
**Q14**	**Medium for storage of avulsed teeth**	212 (37.1)	188 (32.9)	171 (29.9)

For Q9–12, 13i & 13ii, if more than one option, including an incorrect one, were chosen, the answer is considered as incorrect.

For Q14, 1 or more options could be chosen. If any incorrect option is chosen, the question is considered as incorrect.

The total scores are shown in Table 5. The mean of the total score was negative, with value −0.17. The median was 0. It showed that secondary school students' knowledge of dental injury treatment was insufficient.

**Table 5:** Summary statistics of the total score of the respondents.

Minimum	−9
Maximum	8
Mean	−0.17
Median	0
Standard Deviation	2.75

From Question 9 – 13, 1 mark is awarded if only the correct answer was chosen, 0 for don’t know and −1 if any wrong answer was chosen (range: −6 to 6).

For question 14, 1 for each correct answer, 0 for don’t know, −1 for each wrong answer (range: −7 to 3).

Range of total score of the whole questionnaire: −13 to 9.

The regression results in Table 6 indicate that demographic background including gender, age, and form did not have significant effects on the knowledge of dental injury treatment. *X_5_* was significant, i.e., first-aid training without dental injury treatment education would significantly increase the total score (p-value  = 0.0344). On the contrary, *X_6_* was not significant, which implied that students with first-aid training along with dental injury management did not significantly respond to the questions better than the rest. Confidence in distinguishing the type of teeth was not significant. On the other hand, acquisition of dental injury information from other sources was a significant factor that led to higher scores (p-value  = 0.0407).

**Table 6:** Relationship between total score and the independent variables.

	Estimate	Standard Error	p-value
**Intercept**	−0.9175	0.6355	0.1552
**Gender** (0 = Male, 1 = Female)	0.4448	0.2429	0.0677
**Age** **	−0.2413	0.2709	0.3743
**Form**	0.5280	0.2939	0.0755
**First-aid training and dental injury content**			
**X_5_** * (0 = No first-aid training, 1 = First-aid training without dental injury content)	0.9727	0.4586	0.0344
**X_6_** (0 = No first-aid training, 1 = First-aid training with dental injury content)	−0.2393	0.8119	0.7683
**Ability to distinguish the type of teeth** (0 = No, 1 = Yes)	0.4226	0.2732	0.1224
**Receipt of dental injury management information from other sources besides first-aid training** * (0 = No, 1 = Yes)	0.5237	0.2553	0.0407

the independent variable is significantly different from zero at 5% significance

It is coded according to the categorization on the questionnaire, i.e. 1 for 10 or below, 2 for 11 – 13 etc.

## Discussion

This survey recruited randomly selected secondary schools from the list provided by the Educational Bureau. This should be the most accurate and complete list of all secondary schools in Hong Kong available. The School Dental Care Service has been providing basic and preventive dental care to almost all primary school children in Hong Kong since 1980 [27]. All local primary students are eligible to join the School Dental Care Service. Most of them join the School Dental Care Service with guardians' consent. Students cease to be eligible for the School Dental Care Service once they have entered secondary schools.

Since 1994, on signing up for the School Dental Care Service, primary school students would receive a dental handbook, which contains a page on immediate management of avulsion.

The results showed that the knowledge of emergency management of dental trauma of secondary school students in Hong Kong was less favourable than we would like it to be (Table 3, 4, 5). Such results were similar to those of other studies, in which the participants were children and youngsters [15]–[17]. Educational campaign on emergency management of dental trauma can be launched to increase the awareness and level of knowledge of the topic.

A voluntary self-completed, individually responded to questionnaire survey was conducted in the same period of the present study through the Hong Kong Professional Teachers' Union. It showed that primary and secondary school teachers in Hong Kong lacked knowledge of management of traumatic dental injuries. Educational campaign for them was recommended [19].

In general, avulsed permanent teeth should be replanted [21], [24], [25], [28] or should be placed in milk, physiological saline or saliva if immediate replantation is not possible [21], [24], [25], [28]. Deciduous teeth should not be replanted [24]–[26], [28]. In order to carry out the most appropriate emergency management, it is important that the persons at the scene of dental injury are able to distinguish between deciduous and permanent teeth. Given that 76.4% (436/571) respondents stated that they could not distinguish between permanent and deciduous teeth (Table 2), future education materials should include the instruction that an avulsed permanent tooth should be replanted if one feels confident to do so. Otherwise, simply immense the avulsed tooth in milk, physiological saline or saliva if one is not sure about the tooth type or is not confident with the procedure. This makes students be more confident in managing traumatic dental injuries for patients with deciduous, mixed or permanent dentition.

In the regression results shown in Table 6, the demographic background including gender, age, form, and confidence in distinguishing the type of teeth did not have significant effects on the knowledge of dental injury management. *X_5_* was significant, suggesting that first-aid training that did not include dental injury management would significantly increase the total score. Also, prior acquisition of dental injury information from other sources was a significant factor that positive response from these questions would lead to higher scores. *X_6_* was not significant, representing that first-aid training with dental injury treatment education did not significantly affect the total score. However, there were only 17 students who had received first-aid training with dental injury content in the sample, which constituted only a very small proportion. It is likely that this variable did not reach statistical significance because of the small number of such students.

Only 29.8% (170/594) of the students acquired dental injury information from other sources (Table 2). Therefore, it is beneficial to launch educational campaigns in secondary schools to improve students' knowledge of dental trauma.

Only 36.6% (209/571) of the respondents stated that it was correct to go to dentists directly (Table 4) while most respondents thought that doctors and nurses in casualty or in private sector could help the patients (Table 3). Therefore, health educators and doctors should inform students that patients should go to the dentists immediately when they sustain dental injuries. It is also recommended that the two local medical schools and the two universities should include basic management of dental trauma in their undergraduate curricula and continuing professional development programmes of medical and nursing courses.

In the sample, 15.6% of the students thought that it was correct to call an ambulance (Table 3). This message should be conveyed to the Fire Services Department and they should also be advised to train the first aiders in emergency management of dental trauma.

## Conclusion

Hong Kong secondary school students' knowledge of emergency management of dental trauma is insufficient. First-aid training and acquisition of dental injury information from other sources positively correlate with the level of knowledge. Educational campaign on dental trauma management is recommended for secondary schools. We suggest that emergency management of dental trauma should be added to first-aid publications and taught to students and health professionals.

### Follow up action

We prepared a dental trauma emergency management educational poster [29] with one side in Chinese and the other side in English (see Chinese and English educational posters S1 and S2). We have sent 22 copies of this poster to each secondary school in Hong Kong. The poster emphasizes that an injured person should go to a dentist directly. We advised the schools to display the posters on the notice boards of classrooms and at medical rooms. We have sent the posters to the fire service department, all casualties and universities that provide medical and nursing courses in Hong Kong. We have also sent letters to institutions requesting the course coordinators to include emergency management of dental trauma in the undergraduate and the continuing professional development programmes of healthcare professionals.

## Supporting Information

Protocol S1 (PDF)

Chinese Educational Poster S1 (PDF)

English Educational Poster S2 (PDF)
