# Supplementary material for: A Survey on Hong Kong Secondary School Students' Knowledge of Emergency Management of Dental Trauma
Source: PLoS One. 2014 Jan 6;9(1):e84406. doi: 10.1371/journal.pone.0084406 (PMC3882231; doi:10.1371/journal.pone.0084406)
Supplement: Protocol S1 — (PDF) [file pone.0084406.s001.pdf]

香港大學
University of Hong Kong

醫院管理局
HOSPITAL AUTHORITY

香港大學及醫管局港島西醫院聯網研究倫理委員會

Institutional Review Board of the University of Hong Kong/ Hospital Authority Hong Kong West Cluster (HKU/HA HKW IRB)

Address: Rm 901, Administration Block, QMH Tel 2255 3923 2255 4086 Fax 2255 4735

Dr. Cecilia Young
105A, 1/F Liberte Place
833 Lai Chi Kok Rd, Kln

13-May-11

Dear Dr. Young,

IRB Reference Number: **UW 11-186**

The HKU/HA HKW IRB is authorized by a joint agreement of the University of Hong Kong and Hospital Authority Hong Kong West Cluster to review and monitor clinical research. It serves to ensure that research complies with the Declaration of Helsinki and acts in accordance to ICH GCP guidelines, local regulations and Hospital Authority and the University policies.

I write to inform that your research application/submission has been approved by an expedited process with details shown below. You are also requested to adhere to the conditions listed.

**IRB reviewer(s)**	:	Professor CL Lai, Deputy Chairman and Dr. Sydney Tang, Deputy Chairman of the HKU/HA HKW IRB
**Protocol title**	:	A Survey on Knowledge of Emergency Management of Dental Trauma of Secondary students in Hong Kong
**Study site(s)**	:	As stated in application form
**Document(s) approved**	:	01. Clinical research ethics review application form
	:	02. Protocol (SS01 2.4.2011)
	:	03. Information sheet (2.4.2011) - English and Chinese
	:	04. School's informed consent form (2.4.2011) - English and Chinese
	:	05. Guardian's informed consent form (2.4.2011) - English and Chinese
	:	06. Questionnaire - English and Chinese
**Document(s) reviewed**	:	07. Short CV of principal investigator

(Conditions :
1. Do not deviate from, or make changes to the study protocol without prior written IRB approval, except when it is necessary to eliminate immediate hazards to research subjects or when the change involves only logistical or administrative issues.
2. Report the following to HKU/HA HKW IRB: (i) study protocol or consent document change (use 'HKU/HA HKW IRB RE001F7'), (ii) serious adverse event (use 'HKU/HA HKW IRB RE001F8'). (iii) study progress (use 'HKU/HA HKW IRB RE001F9a')* (iv) new information that may be relevant to a subject's willingness to continue participation in the study.
3. Report study progress to HKU/HA HKW IRB at a 12-monthly interval until study closure.)

Yours sincerely.

W. H. Lee
Secretary, HKU/HA HKW IRB

Protocol SS01 2.4.2011

A Survey on Knowledge of Emergency Management of Dental Trauma of Secondary Students in Hong Kong

Background

Secondary students are physically active. Dental injuries are common. It is important to know whether they have the knowledge to take simple but essential emergency steps before they reach the professional care.

There is no information of knowledge of emergency management of dental trauma of secondary students in Hong Kong

The questionnaire is constructed according to the principle that only questions related to the most critical and essential knowledge should be asked.

Aim

To investigate the knowledge of emergency management of dental trauma of secondary students in Hong Kong

Methodology

A questionnaire survey of secondary students in terms of randomly selected schools will be conducted.

Design

A questionnaire is designed. An invitation letter with school consent form and individual guardian's consent form will be sent to each randomly selected school. The list of schools was given by the educational bureau through written request. At 40 students are needed per participating schools.

This study will comply with Declaration of Helsinki and ICH GCP guidelines.

Eligibility

All secondary schools in Hong Kong that the students can read Chinese or English.

Withdrawal of students from the study

The participating students can withdraw from the study at any time.

Sample size determination:

For the calculation of sample size, we use a confidence level of 95% and a precision of 10%. As the population variance is unknown, we adopt P = 50% ensure the greatest variance will be resulted. Assuming an infinite population size, we need a sample of size 97. To account for the design of a cluster sampling, we multiply a design effect of 1 + (m-1)ρ, where m is the cluster size and ρ is the intracluster correlation (ICC). We assume a cluster size of 40 and ICC of 0.1. No published data on ICC under this setting can be found. However, in general practice studies, ICC take values commonly between 0.01 and 0.05 [1], so 0.1 would be a conservative estimate. We have a sample size of 476, or a total of 12 schools. To account for potential dropout, blank or incomplete returned questionnaire, we recruit an extra 20%, which results in 15 schools, or 600 students.

Recruitment

An invitation letter will be sent to each randomly selected school from the list of secondary schools given by the Educational Bureau. A school consent form and individual guardian's consent form will also be sent together. The filled questionnaire will be collected through the schools.

Sample in terms of school as a unit is recruited. At least 40 students per participating school are needed. Until the sample size is reached.

Statistical Analysis

For each question concerning knowledge about dental trauma, we calculate the number and proportion of respondents who choose the correct answer(s). We also calculate the proportion of students reaching a certain level of knowledge by evaluating their total scores. It would reflect the knowledge of general students. We would also study the associations between factors like student's gender, age, form, whether he/she has received first aid training etc and their scores. Linear regression would be conducted to evaluate that which factors would affect the scores significantly.

Reference

1. Underwood M, Barnett A, Hajioff S. *Cluster randomization: a trap for the unwary.* Br J Gen Pract 1998; 48: 1089-1090.

Dear Principal, PTA Chairperson, teachers and guardians,

Ref: Academic Study for dental injuries – questionnaire survey for students

I am a public health practitioner and researcher, I am now co-operating with Prof Cheung Lim Kwong and Mr Alex Wong in an academic study for dental injuries. This is a joint project with the University of Hong Kong. We invite your school to join this study.

The aim is to investigate the knowledge about dental injuries of secondary students in Hong Kong. The questionnaire consists of 14 MC questions which can be filled in around **7 minutes**. They are basic data and dental injury knowledge questions. There will be no sensitive questions and all information will comply with rules in Hong Kong and international medical research ethics code.

We will send an educational poster to all participating schools after the survey. Students of the participating schools will be the first group to learn the essential but simple and precise steps of how to deal with dental injuries in accidents before professional care are provided. This will benefit your students and save more teeth.

The result of this study will give some information to the field. If the survey can be completed, all secondary schools in Hong Kong will receive the same educational poster.

We cannot proceed to the printing and sending of the posters to other secondary schools without this study. Your participation is important to all secondary schools. We will acknowledge all participating schools in a list when we send the posters. Please tell us if you do not want your school appears in the list.

We gave the guardian's consent forms, questionnaires and posters to some F.1 students and their guardians; they filled out, returned and told us that this topic was highly related to their daily life. They hope this study could be completed and educational posters could be sent to all secondary schools in Hong Kong.

Please sign the **school consent form** and **individual guardian's consent form** if you can help. **However, students have the absolute right to withdraw before they send the filled questionnaire even they signed the consent form.** We need at least 40 students in your school.

We need your support. Please discuss with your collegues and guardian's representatives, kindly contact Cecilia Young at 23618684 or ceciliatyp@yahoo.com.hk if you have any enquiries. Please mail the **consent forms** and the filled questionnaires with the **prepaid freepost envelope**. Please contact Cecilia Young immediately if you do not join us, we need to randomly select another school to replace your school immediately.

Thank you for your kind attention.
Cecilia Young

Research Team – Survey – Dental Injuries

105A, 1/F Liberte Place, 833 Lai Chi Kok Road, Kln Tel 23618684 ceciliatyp@yahoo.com.hk

1.7.2011

親愛的校長，家長教師會主席，老師及家長：

醫學研究 — 牙齒創傷

香港大學的張念光教授及黃堅祐先生與我現進行牙齒創傷的醫學調查，我們邀請貴校參與。

是次研究目的為調查香港中學生在牙齒創傷方面的知識，有十四條選擇題，大約需要七分鐘，內容為基本資料及牙齒創傷問題，不涉及敏感問題，並會依從所有香港法例及國際性的醫學研究倫理守則處理（包括保密及其他）。

完成後，我們會首先寄出教育性海報給參與的學校，參與的學生是全港第一批接觸有關牙齒創傷即時處理方法的中學生，內容包括傷者於正式醫治前簡單而必需的處理步驟，正確方法能救回更多牙齒。

本研究將給予牙醫學界一些數據，如調查順利完成，全港所有中學都會收到相同教育性海報。

印制及寄出海報給全港中學需以此調查結果為基礎，如此調查不能完成，我們無法寄出海報給其他學校，貴校的參與對所有中學十分重要。我們在寄出海報時會鳴謝參與的學校，如不希望列出貴校，請告知我們。

我們曾把學生家長同意書、問卷及海報給予一些中一學生及監護人，他們均認為牙齒創傷資料對日常生活有用，希望調查順利完成，全港中學都收到此海報。

請參與本調查，填寫校方同意書及學生家長同意書(18 歲以上學生請列明歲數，不用家長簽署) 。**但參與學生即使已簽署家長同意書，在交問卷前仍有絕對權利退出調查。**每間參與學校最少需要 40 名學生。

每間參與學校**需要最少 40 名學生**。請校長或負責老師隨機抽樣任何一至數班同學 (如一班不足，便邀請兩班，多於 40 人即可)。

我們需要您的支持! 請參與是次調查。敬希盡早以 **freepost** 回郵信封寄回一份**校方同意書**，所有參與學生的**家長同意書及填妥問卷** (請填寫學生正楷姓名)，**暑假前最佳**，如認為九月中開學忙碌過後才有時間進行，需更多時間或有查詢請電 23618684 或電郵 ceciliatyp@yahoo.com.hk 楊小姐。

如未能參與，請立即致電 23618684 或 電郵 ceciliatyp@yahoo.com.hk 楊小姐，我們需即時隨機抽樣，邀請另一學校代替。請寄回所有文件。謝謝！

研究團隊　　　　　　　　　　　　(所有文件已經寄給校長)

School's Informed Consent Form

A Survey on knowledge of emergency management of dental trauma of Secondary Students in Hong Kong

1. The aim of this study is to investigate the knowledge about dental injuries of secondary students in Hong Kong.

2. The questionnaire consists of 14 multiple questions in the questionnaires. They are basic data and dental injury knowledge. There will be no sensitive questions and all information will comply with all rules in Hong Kong (keep confidential and other law) and international medical research ethics code.

3. Participating students are the first group of secondary students who receive simple and basic guidelines of handling of dental injuries in an educational poster..

4. The data will give some information to the field. If the study is completed, educational posters will be sent to all secondary schools in Hong Kong. The printing and sending can only be proceeded with the basis of this study.

5. **Participating student has the absolute right to withdraw this study at any stage.**

6. A copy of this consent form will be given to the school.

Participating school: ______________________________

Number of classes:

Person in charge of this project (print name):

Email: ____________________ Tel:

Date:

____

Authorized signature with school chop

校方知情同意書

中學生對牙齒創傷知識調查

1. 調查目的是研究香港中學生對牙齒創傷的認識。

2. 問卷有十四條選擇題。內容為基本資料及牙齒創傷問題，不涉及敏感資料，並會依從所有香港法例及國際性的醫學研究倫理守則（包括保密及其他）。

3. 完成後，會首先寄出教育性海報給參與的學校，參與的學生是全港第一批接觸有關牙齒創傷即時處理方法的中學生，內容包括傷者於正式醫治前簡單而必需的處理步驟

4. 調查結果用作學術研究用途。本研究將給予牙醫學界一些數據，最後全港所有中學均會收到牙齒創傷知識資料。印刷及郵遞海報給其他學校必需在研究完成後進行並以此為基礎。

5. **參與學生即使已簽署同意書仍有絕對權利退出研究。**

6. 本同意書副本將給予校方。

參與學校： ______________________________

數目： _____ 班

負責人 (正楷)： ______________ **電郵** ______________________

電話　： ________________

日期　： ________________

____

校方簽署及蓋印

Guardian's Informed Consent Form (no need to fill if aged 18 or above)

A Survey on knowledge of emergency management of dental trauma of Secondary Students in Hong Kong

1. The aim of this study is to investigate the knowledge about dental injuries of secondary students in Hong Kong.

2. The questionnaire consists of 14 multiple questions in the questionnaires. They are basic data and dental injury knowledge. There will be no sensitive questions and all information will comply with all rules in Hong Kong (keep confidential and other law) and international medical research ethics code.

3. Participating students are the first group of secondary students who receive simple and basic guidelines of handling of dental injuries in an educational poster..

4. The data will give some information to the field. If the study is completed, educational posters will be sent to all secondary schools in Hong Kong. The printing and sending can only be proceeded with the basis of this study.

5. **Participating student has the absolute right to withdraw this study at any stage.**

6. A copy of this consent form will be given to each participating student's guardian.

I can read English.

Student's Print Name: **Class:**

School: ______________________________

Please give Chinese / English questionnaire to student.
(Please cross out the unsuitable language)

Guardian's Print Name:

____
Signature
Date:

學生家長知情同意書 (如學生年滿 18 歲, 不用簽署本同意書，請在問卷上列明已年滿 18 歲)

香港中學生對牙齒創傷知識調查

1. 調查目的是研究香港中學生對牙齒創傷的認識。

2. 問卷有十四條選擇題。內容為基本資料及牙齒創傷問題，不涉及敏感資料，並會依從所有香港法例及國際性的醫學研究倫理守則（包括保密及其他）。

3. 完成後，會首先寄出教育性海報給參與的學校，參與的學生是全港第一批接觸有關牙齒創傷即時處理方法的中學生，內容包括傷者於正式醫治前簡單而必需的處理步驟

4. 調查結果用作學術研究用途。本研究將給予牙醫學界一些數據，最後全港所有中學均會收到牙齒創傷知識資料。印刷及郵遞海報給其他學校必需在研究完成後進行並以此為基礎。

5. 參與學生即使已簽署同意書仍有絕對權利退出研究。

6. 本同意書副本將給予學生家長。

本人能閱讀中文。

參與學生姓名(正楷): ______________ 班別及學號(必須填寫) ________ :

學校: ________________________________

給學生問卷請用 中文/英文 (請刪去不適用語言)

家長或監護人姓名(正楷): ________________________

____

家長或監護人簽署

日期:

各位同學，18 歲以下必須交回已填簽署了的家長同意書，姓名或班別學號應與家長同意書一樣，入電腦後沒有姓名，只有編號 ST001 –ST600。

醫學用問卷資料，18 歲以下沒有家長同意書不能使用。所以我們必須對以下資料與家長同意書一致。

○ 本人已滿 18 歲，不用交家長同意書 ______________ (學生必須簽署)
○ 本人未滿 18 歲，已交簽署好的家長同意書，請對以下姓名或班別學號

同學姓名(正楷)：______________ 班別學號(必須填寫)：__________
學校 ：________________________________
日期 ：__________________

香港中學生對牙齒創傷知識調查

第一部份 基本統計資料

請在適當 ○上填上✓號。

1. 性別 ： ○ a. 男 ○ b. 女

2. 年齡 ： ○ a. 十歲或以下 ○ b. 十一至十三歲 ○ c. 十四至十六歲 ○ d. 十七至十九歲 ○ e. 二十歲或以上

3. 班級 ： ○ a. 中一至中三 ○ b. 中四至中五 ○ c. 中六至中七

4. 你曾否接受過正規急救訓練？
○ a. 有, 請列明 ___________ (往第 5 條) ○ b. 沒有 (往第 6 條)

5. 正規急救課程內容有否包括牙齒創傷？
○ a. 有 ○ b. 沒有

6. 你認為自己是否能從傷者年齡，觀察受傷牙齒外形、牙床及其位置分辨出乳齒或恆齒？
○ a. 是 ○ b. 不是

7. 除正式急救外，在這次調查以前，你曾否閱讀或收聽過任何有關牙齒創傷的教育資料？

○ a. 有 （往第 8 條） ○ b. 沒有（往第 9 條）

8. 你從哪種途徑得悉有關牙齒創傷的教育資料？

○ a.電視 ○ b.雜誌 ○ c.電台 ○ d.學校
○ e.海報 ○ f.報紙 ○ g.互聯網 ○ h.口頭描述
○ i.其他（請列明）____________________

第二部份 有關知識

9. 如果有意外，以致牙齒創傷，其受傷位置只在牙齒及附近口腔位置，傷者應怎樣做？

○ a. 步行或以交通工具往最近的醫院急症室
○ b. 召救傷車，往最近醫院急症室
○ c. 往最近的私人執業全科醫生
○ d. 往傷者的家庭醫生
○ e. 往牙醫
○ f. 自行處理
○ g. 其他（請列明）____________________
○ h. 不知道

10. 傷者應何時前往或被送往診治？

○ a. 午膳或放學後
○ b. 充分休息後
○ c. 二十四小時內
○ d. 四十八小時內
○ e. 當家長或監護人有空帶傷者前往診治時
○ f. 當傷者放鬆心情並希望診治時
○ g. 四小時內
○ h. 立刻
○ i. 其他（請列明）____________________
○ j. 不知道

11. 如牙齒斷裂，傷者或附近人士應：

○ a. 斷裂部份無用，不需理會
○ b. 找出斷裂部份，以紗布或紙巾包裹帶往診治
○ c. 把斷裂部份放入液體內，帶往診治
○ d. 其他（請列明）____________________
○ e. 不知道

12. 如牙齒依然留在牙床內但移位或拉出了一些，傷者或附近人士應：

○ a. 不應接觸，保持現在新的位置
○ b. 嘗試用手指把它移回原位
○ c. 如有可能，請傷者小心慢慢地咬合
○ d. 其他（請列明）____________________
○ e. 不知道

13. 碰撞後飛脫的牙齒是否應該放回牙床原來位置？

乳齒	○ a. 應該	○ b. 不應該	○ c. 不知道
恆齒	○ a. 應該	○ b. 不應該	○ c. 不知道

14. 如牙齒碰撞後飛脫，應：（可選擇一個或以上）

○ a. 牙齒沒有用處，不應浪費時間找尋及處理
○ b. 以紗布或紙巾包裹，帶往診治
○ c. 放入凍牛奶內，浸過牙齒，帶往診治
○ d. 放入生理鹽水內（從藥房買來的），浸過牙齒，帶往診治
○ e. 放入盛有傷者的口水的器皿內，浸過牙齒，帶往診治
○ f. 放入盛有自來水的器皿內，浸過牙齒，帶往診治
○ g. 放入盛有蒸餾水的器皿內，浸過牙齒，帶往診治
○ h. 放入器皿或膠袋內，乾的狀態下，帶往診治
○ i. 放入盛有消毒藥水的器皿內，浸過牙齒，帶往診治
○ j. 其他（請列明）____________________
○ k. 不知道

問卷完

We need to check the Guardian's consent if student is under 18 year old. This is because we cannot use the information for medical research without it.

Please make sure you have given your guardian consent form to your teacher and fill the following information for checking.

Name: ____________________ **Class and class number (necessary):** _________
School: ________________________
Date:

You do not need to fill the guardian consent form if you are 18 or above.
○ I am aged 18 or above. Name/ Class number (necessary): _____________

A Survey on knowledge of emergency management of dental trauma of Secondary Students in Hong Kong

Part 1 demographics Please put a ✓ in your answer.

1. Gender: ○ a. Male ○ b. female

2. Age: ○ a. under 10 years old ○ b. 11-13 years old ○ c. 14-16 years old
○ d. 17-19 years old ○ e. 20 or above

3. Which grade are you studying?
○ a. F.1-F.3 ○ b. F.4-F.5 ○ c. F.6 – F.7

4. Have you received any formal first aid training?
○ a. Yes, please specify __________ (go to Q5) ○ b. No (go to Q6)

5. Is the first aid about dental injury included in your formal first aid training?
○ a. Yes ○ b. No

6. Do you think that you can distinguish a primary tooth (baby tooth) from a permanent tooth by considering the patient's age and looking at the tooth, the socket or the position?
○ a. Yes ○ b. No

7. Have you ever received any educational material/information about the injury except formal first aid training (before this study)?
○ a. Yes (go to Q.8) ○ b. No (go to Q.9)

8. Which way?

○ a. TV ○ b. Journals ○ c. Radio ○ d. School
○ e. Poster ○ f. Newspapers ○ g. Internet ○ h. Oral description
○ i. Others (please specify) ____________________

Part II knowledge

9. If there is a dental injury, the impact sites are the teeth, only the teeth and surrounding part seemed to be injured, what should the patient do?

○ a. Go to the casualty in the nearest hospital on foot or by any transport
○ b. Call an ambulance; go to the casualty in the nearest hospital.
○ c. Go to the nearest private doctor
○ d. Go to the patient's family doctor
○ e. A dentist
○ f. Treat it by self
○ g. Others (please specify)____________________
○ h. Don't Know

10. When should the patient go or be brought for the examination and treatment?

○ a. At lunch or after school
○ b. After plenty of rest
○ c. Within 24 hours
○ d. Within 48 hours
○ e. When the parent or guardian is free to bring the patient for examination and treatment
○ f. Any time when the patient feels relax and want to have treatment
○ g. Within 4 hours
○ h. Immediately
○ i. Others (please specify) ____________________
○ j. Don't Know

11. If the tooth is fractured, the patient or surrounding person should:

○ a. The fractured part is useless, ignore it
○ b. Try to find the fractured part, wrap it with gauze or tissue and bring it for examination and treatment
○ c. Put it in liquid medium and bring it for examination and treatment
○ d. Others (please specify) ____________________
○ e. Don't Know

12. If the tooth is displaced or extruded but still attached, the patient or surrounding person should:

- ○ a. Do not touch, let it remains its new position
- ○ b. Try to put back to the original position
- ○ c. Ask the patient to carefully clench one's teeth if it is possible
- ○ d. Others (please specify) ___________________
- ○ e. Don't Know

13. Should a knocked out tooth be put back to the original position?

Baby tooth	○ a. Yes	○ b. No	○ c. Don't know
Permanent tooth	○ a. Yes	○ b. No	○ c. Don't know

14. If a tooth is knocked out? It should be: (1 or more)

- ○ a. The tooth is useless, do not spend time to find it or to work on it
- ○ b. Wrap it in gauze or tissue and bring it to examination and treatment
- ○ c. Soak it in cold milk in a container and bring it to examination and treatment
- ○ d. Soak it in physiological saline in a container (from pharmacy) and bring it to examination and treatment
- ○ e. Soak it in the patient's saliva in a container and then bring the container to examination and treatment
- ○ f. Soak it in tap water in a container and bring the container to examination and to treatment
- ○ g. Soak it in distilled water in a container and bring the container to examination and to treatment
- ○ h. Put it in a container or a plastic bag in dry condition and bring it to examination and treatment
- ○ i. Soak it in a container with disinfectant solution and bring it to examination and treatment
- ○ j. Others (please specify) ___________________
- ○ k. Don't Know

End of the questionnaire
